# Giant coronary artery aneurysms in juvenile polyarteritis nodosa: a case report

**DOI:** 10.1186/1546-0096-10-1

**Published:** 2012-01-05

**Authors:** Therese L Canares, Dawn M Wahezi, Kanwal M Farooqi, Robert H Pass, Norman T Ilowite

**Affiliations:** 1 Department of Pediatrics, Children's Hospital at Montefiore, 3415 Bainbridge Ave, Bronx, NY, 10467, USA; 2 Department of Pediatrics, Division of Rheumatology, Children's Hospital at Montefiore, 3415 Bainbridge Ave, Bronx, NY, 10467, USA; 3 Department of Pediatrics, Division of Cardiology, Children's Hospital at Montefiore, 3415 Bainbridge Ave, Bronx, NY, 10467, USA

**Keywords:** polyarteritis nodosa, vasculitis, coronary artery aneurysm, Kawasaki disease

## Abstract

Juvenile polyarteritis nodosa (PAN) is a rare, necrotizing vasculitis, primarily affecting small to medium-sized muscular arteries. Cardiac involvement amongst patients with PAN is uncommon and reports of coronary artery aneurysms in juvenile PAN are exceedingly rare. We describe a 16 year old girl who presented with fever, arthritis and two giant coronary artery aneurysms, initially diagnosed as atypical Kawasaki disease and treated with IVIG and methylprednisolone. Her persistent fevers, arthritis, myalgias were refractory to treatment, and onset of a vasculitic rash suggested an alternative diagnosis. Based on angiographic abnormalities, polymyalgia, hypertension and skin involvement, this patient met criteria for juvenile PAN. She was treated with six months of intravenous cyclophosphamide and high dose corticosteroids for presumed PAN related coronary vasculitis. Maintenance therapy was continued with azathioprine and the patient currently remains without evidence of active vasculitis. She remains on anticoagulation for persistence of the aneurysms. This case illustrates a rare and unusual presentation of giant coronary artery aneurysms in the setting of juvenile PAN.

## Background

Juvenile polyarteritis nodosa (PAN) is a rare, necrotizing vasculitis, primarily affecting small to medium-sized muscular arteries [1]. Recent classification criteria from EULAR/PRES require histopathology of necrotizing vasculitis or angiographic abnormalities (aneurysm, stenosis, or occlusion of small-medium arteries), plus one of five of the following: skin involvement, myalgia or muscle tenderness, hypertension, peripheral neuropathy, or renal involvement, for diagnosis [2]. Cardiac involvement amongst patients with PAN is not common. Well described cardiac manifestations in adults with PAN include pericarditis, arrhythmia and valvular disease. There are infrequent reports of coronary arteritis, stenosis [3], dissection [4], and occasionally aneurysms [5] associated with PAN, however renal and gastrointestinal aneurysms are more common [6,7]. There is a paucity of literature regarding coronary artery manifestations in juvenile PAN, with limited information on demographics, clinical characteristics, treatment, and outcomes. Herein we describe a case of coronary artery aneurysms in an adolescent with juvenile PAN.

## Case Presentation

This is a report of a 16 year old girl from St. Croix, U.S. Virgin Islands, who had been previously diagnosed with juvenile idiopathic arthritis (JIA) at 5 years of age, based on reports of fever, arthritis, and chronic uveitis. She received no systemic immunosuppressant medication and appeared to be in remission for several years. In December 2009 she presented with three days of sharp, substernal chest pain, two days of fever, and one day of bilateral hip pain causing difficulty ambulating. Physical exam revealed a tall, slender girl with mild nasal congestion. On cardiac exam she had a regular rate and rhythm, normal S1 and S2, and no appreciable murmur. Examination of her extremities revealed tenderness over her bilateral greater trochanters and bilateral knee effusions. Initial laboratory testing demonstrated white blood cell count of 5.7 k/μl, hemoglobin 11.4 g/dl, hematocrit 35.5%, platelets 156 k/μl, erythrocyte sedimentation rate 75 mm/hr, C-reactive protein 16.9 mg/dL, and normal urinalysis, creatinine, and cardiac enzymes. In addition, the patient had a negative rheumatoid factor, HLA-B27, anti-streptolysin O (ASLO), anti-nuclear antibody (ANA); as well as negative anti-cardiolipin, anti-DNA, anti-smith, anti-RNP, anti-SSA, anti-SSB, and anti-neutrophil cytoplasmic antibodies. Ophthalmologic exam performed during hospitalization revealed trace asymptomatic uveitis.

**Figure 1:**
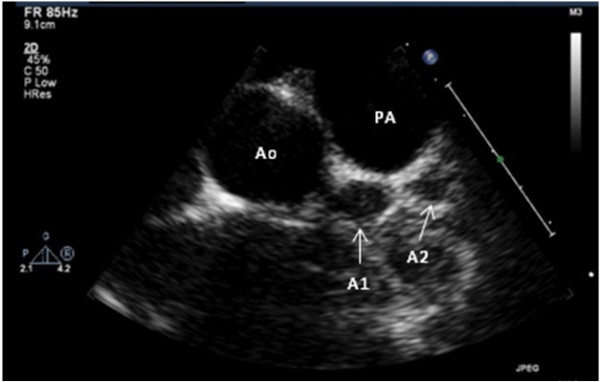
**Aneurysms visualized by echocardiography**. Echocardiographic parasternal short axis view reveals two fusiform aneurysms of the left anterior descending coronary artery. The proximal aneurysm (A1) measures 8.7 mm, and the distal (A2) measures 9.0 mm. The aorta (Ao) and main pulmonary artery (PA) are seen in cross section.

Due to the severity of her chest pain, an echocardiogram was performed, which revealed two giant coronary artery aneurysms. The proximal aneurysm involved the left main coronary artery and measured 9 mm. The second aneurysm involved the left anterior descending artery distally and measured 8.7 mm (Figure 1). She had qualitatively normal biventricular systolic function. Radiographic imaging using magnetic resonance angiography and doppler ultrasound revealed no other vascular abnormalities in the chest, abdomen, pelvis, renal and carotid arteries. Due to a prolonged course of 8 days of fevers, the presence of the coronary aneurysms and a concern for atypical Kawasaki disease (KD), the patient was started on aspirin and received two courses of IVIG. In addition, she was started on anticoagulation with warfarin. She was additionally treated with 1 gram of intravenous methylprednisolone due to evidence of persistent inflammation and her fever subsequently resolved.

One week following a ten day hospitalization, the patient developed recurrence of fevers, arthritis and elevation of acute phase reactants. Due to concern for active systemic vasculitis, she was given a subsequent dose of 1 gram of intravenous methylprednisolone, followed by high dose oral corticosteroids (prednisone 60 mg daily). Gallium scan was performed to evaluate for sarcoidosis and revealed no evidence of abnormal Gallium uptake. Despite high dose corticosteroids, the patient continued to have persistent fevers, arthritis, myalgias, and elevated acute phase reactants, and developed a new erythematous, palpable, non-blanching purpura over bilateral lower extremities. Skin biopsy demonstrated leukocytoclastic vasculitis. Two weeks after her rash appeared, she additionally developed persistent hypertension, requiring treatment with amlodipine. This patient met criteria for juvenile PAN based on angiographic abnormalities and myalgias. This diagnosis was further supported by her hypertension and vasculitic rash. She was treated with six months of intravenous cyclophosphamide and high dose corticosteroids for presumed PAN related coronary vasculitis.

**Figures 2:**
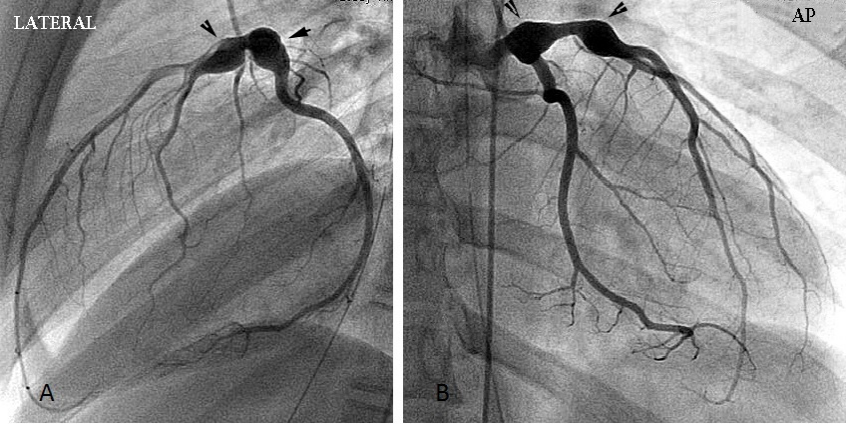
**A and B. Aneurysms revealed by angiography**. Lateral and AP views of selective angiography into the left coronary artery which reveals two aneurysms (arrows). The proximal aneurysm is of the left main coronary artery and the distal aneurysm is of the left anterior descending artery.

During outpatient evaluation, the patient underwent a cardiac catheterization to evaluate distal coronaries and any additional changes that may not have been visualized on echocardiography. On selective coronary angiography, the two aneurysms were confirmed with no additional abnormalities identified (Figure 2). No intervention was made during the catheterization. Additionally, the patient had a single photon emission computed tomography (SPECT) myocardial perfusion scan with a Tc-99 m Sestamibi injection to assess for possible cardiac ischemia which may present during exertion. During the test, she had no chest pain, and there were no electrocardiogram changes suggestive of ischemia. Subsequent echocardiograms, one year following diagnosis, have shown no significant change in the size of the coronary aneurysms nor have new aneurysms been noted. Currently acute phase reactants have normalized and the patient remains with no evidence of active vasculitis. She was started on azathioprine for maintenance therapy and is slowly being tapered off of corticosteroids. She remains on anti-coagulation with warfarin and aspirin.

## Discussion

This case illustrates a rare and interesting cardiac manifestation of juvenile PAN. The presence of giant coronary artery aneurysms led to initial suspicion and treatment for atypical KD. The patient was refractory to IVIG and corticosteroids, as evidenced by persistent vasculitic symptoms, suggesting alternate diagnoses. Based on angiographic abnormalities, myalgias, hypertension and skin involvement, this patient met criteria for juvenile PAN. It is unclear to the authors whether or not this patient's prior diagnosis of JIA was accurate or if she may have had an underlying form of vasculitis from the onset.

KD and PAN have overlap of symptomatology. Both entities are vasculitides affecting small and medium vessels in children [8]. Early in the course either disease can have non-specific signs and symptoms such as fever or malaise. Common signs and symptoms include rash, petechiae, conjunctivitis, arthritis, aneurysms, or abdominal complaints, including pain, emesis, or diarrhea [9]. A number of children have been diagnosed with KD, to later manifest as PAN [8-10]. Similarly, "infantile PAN", historically thought to be within the spectrum of PAN, is now understood to be KD [11,12]. Given that coronary artery aneurysms develop in 17% of untreated KD, it is unsurprising that PAN is mistaken for KD [13]. Coronary aneurysms occur in diseases other than KD, and clinicians should have a higher index of suspicion for other pediatric vasculitides.

Children with PAN may have a wide variety of cardiac manifestations. In a case series of 31 children with PAN, five children demonstrated cardiac involvement including pericarditis, arrhythmia, and cardiac failure. One child died of cardiac failure and hypertension. These patients were treated with corticosteroids with some receiving a cytotoxic agent [14]. Changes on echocardiogram include mitral stenosis, mitral regurgitation, tricuspid regurgitation, mitral valve prolapse, and pericardial thickening [15-17]. Rare case reports additionally describe electrocardiographic changes, cardiomegaly, myocarditis, myocardial fibrosis, endocarditis, coronary arteritis, myocardial infarction, pericarditis, and hypertrophy [3,17,18]. Histopathology has demonstrated evidence of cardiac involvement with coronary arteritis found in 18/36 cases of necrotizing vasculitis on autopsy [19].

Detailed accounts of coronary artery involvement in juvenile PAN are even sparser. One case series from Saudi Arabia of children aged 2-11.5 years describes one child with heart failure and coronary artery aneurysms on angiography. The age, clinical characteristics, treatment, and outcome are not included [20]. One of the largest multicenter surveys of juvenile polyarteritis describes 63 patients with systemic PAN. One patient in this group had a cardiac aneurysm, but no further details are provided [7]. In another report, two patients, ages 3 and 15, were found with coronary artery aneurysms and a presumed diagnosis of KD. Both patients were later diagnosed with polyarteritis based on clinical markers, but biopsy confirmation was not described [10].

Treatment of juvenile PAN is primarily aimed at decreasing systemic vascular inflammation. It has been demonstrated that corticosteroid therapy has an essential role in the initial management, with second line therapies including cyclophosphamide or azathioprine [7,21]. Additional reports suggest successful outcomes with methotrexate, mycophenolate mofetil, IVIG, infliximab, or rituximab, however these reports are largely based on adult-data, case reports and retrospective reviews [21]. Anti-coagulation with warfarin and aspirin was used to prevent clot formation in our patient's coronary artery aneurysms. Due to the paucity of information on coronary artery aneurysms in PAN, treatment of her aneurysms was extrapolated from experience with KD and other systemic vasculitides. In KD, treatment for persistent aneurysms is dependent on the size and flow of the lesion. The treatment of giant aneurysms (≥8 mm) with sluggish flow may consist of systemic anticoagulation in addition to anti-platelet therapy [22]. Those patients who are asymptomatic with smaller residual aneurysms are to be continued on aspirin and make improvements in their lifestyle such as weight management in obese patients. In patients with symptoms suggestive of ischemia, however, other therapies must be initiated and testing done to identify possible worsening of their clinical status [23]. An invasive intervention may be necessary in the symptomatic patient. In the pediatric population, 'infantile PAN' or KD, associated with coronary aneurysms has been described with recovery and even regression of the aneurysms [24,25]. Outcomes of coronary artery aneurysms in PAN identified pre-mortem are not well described.

## Conclusion

Herein we report a rare case of an adolescent with juvenile PAN and multiple giant coronary artery aneurysms. Medical management with intravenous pulse cyclophosphamide and high dose corticosteroids led to resolution of all evidence of active vasculitis. Currently, our patient's giant coronary aneurysms remain asymptomatic and she continues on maintenance therapy with azathioprine, warfarin and aspirin.

## Consent

Written informed consent was obtained from the patient's parent for publication of this case report and any accompanying images.

## Abbreviations

PAN: polyarteritis nodosa; KD: Kawasaki disease

## Competing interests (Financial or Non-Financial)

The authors declare that they have no competing interests.

## Authors' contributions

TC reviewed medical records, gathered patient data, and primarily authored this manuscript. DW conducted literature review and authored sections pertaining to pediatric rheumatology. KF conducted literature review and authored sections pertaining to pediatric cardiology. RP provided images of cardiac catheterization and edited sections pertaining to pediatric cardiology. NI provided critical revision and editing of this manuscript. All authors read and approved the final manuscript.

## Author's Information

NI is the Chief of Pediatric Rheumatology and a Professor of Pediatrics at The Children's Hospital at Montefiore, Albert Einstein College of Medicine. RP is the Director of Pediatric Electrophysiology, Director of Pediatric Interventional Cardiology, and an Associate Professor at The Children's Hospital at Montefiore, Albert Einstein College of Medicine.
